# Supplementary material for: Multiplex Soluble Biomarker Analysis from Pleural Effusion
Source: Biomolecules. 2020 Jul 28;10(8):1113. doi: 10.3390/biom10081113 (PMC7464384; doi:10.3390/biom10081113)
Supplement: Supplementary file 1 [file biomolecules-10-01113-s001.pdf]

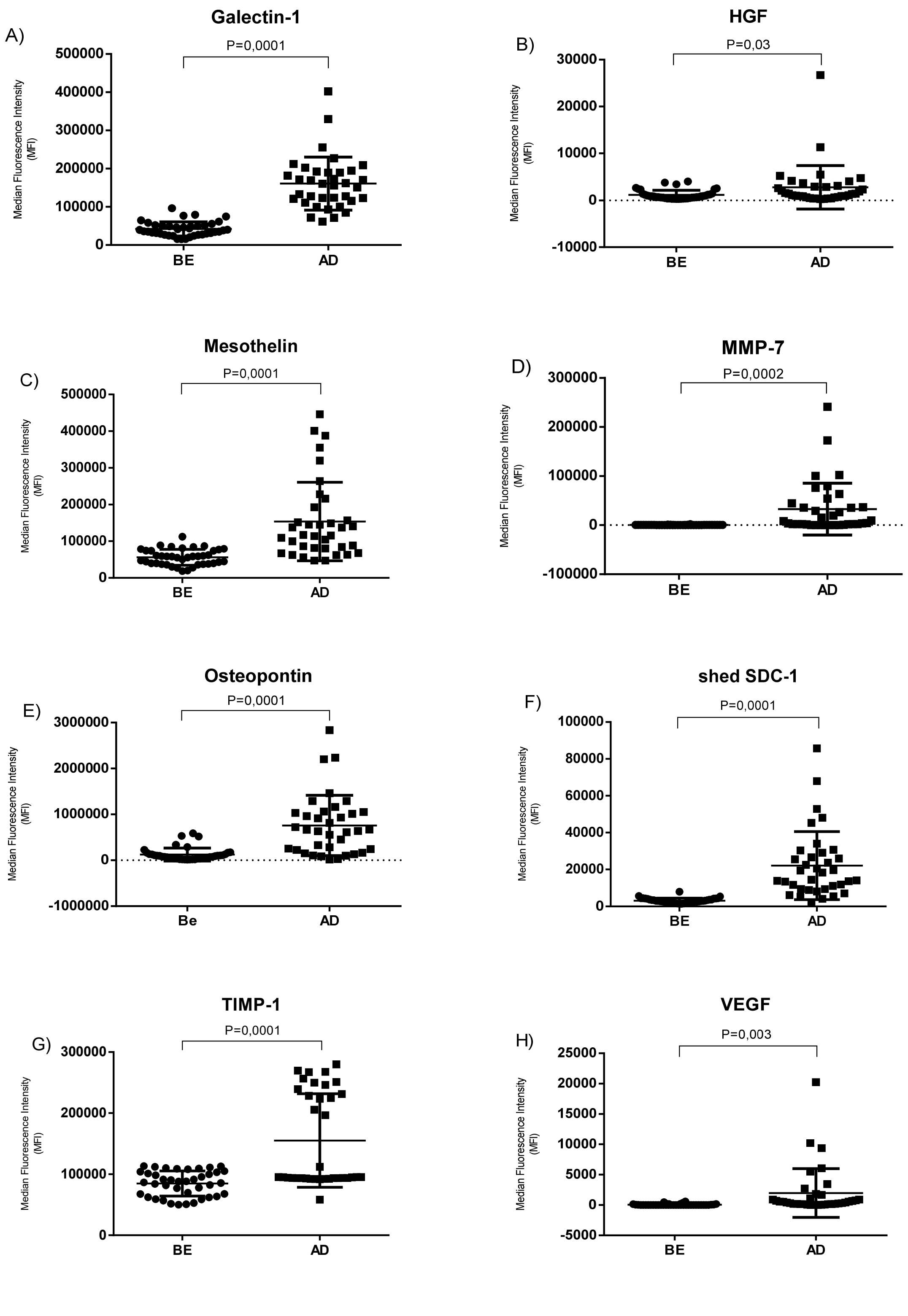
A)
Galectin-1
P=0,0001
Median Fluorescence Intensity (MFI)
500000
400000
300000
200000
100000
0
BE
AD
B)
HGF
P=0,03
Median Fluorescence Intensity (MFI)
30000
20000
10000
0
-10000
BE
AD
C)
Mesothelin
P=0,0001
Median Fluorescence Intensity (MFI)
500000
400000
300000
200000
100000
0
BE
AD
D)
MMP-7
P=0,0002
Median Fluorescence Intensity (MFI)
300000
200000
100000
0
-100000
BE
AD
E)
Osteopontin
P=0,0001
Median Fluorescence Intensity (MFI)
3000000
2000000
1000000
0
-1000000
Be
AD
F)
shed SDC-1
P=0,0001
Median Fluorescence Intensity (MFI)
100000
80000
60000
40000
20000
0
BE
AD
G)
TIMP-1
P=0,0001
Median Fluorescence Intensity (MFI)
300000
200000
100000
0
BE
AD
H)
VEGF
P=0,003
Median Fluorescence Intensity (MFI)
25000
20000
15000
10000
5000
0
-5000
BE
AD

I)

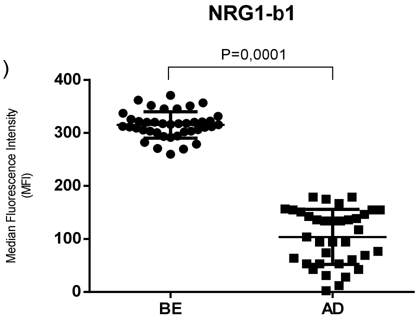

Supplementary Figure 1. Pleural effusion levels of diagnostic biomarkers in metastatic adenocarcinoma patients. Levels of Galectin-1, HGF, Mesothelin, MMP-7, Osteopontin, shed SDC-1, TIMP-1, VEGF are significantly higher in pleural effusion from metastatic adenocarcinoma (AD) patients (n = 36) comparing with benign (BE) patients (n = 40) (**A**–**H**). (**I**). NRG1-β1 has significantly lower level in AD patients comparing with BE. Significance was assessed by two-tailed t-test at $p \leq 0.05$.
